# Pollen Interference Between Rare and Common Species

**DOI:** 10.1002/ece3.70505

**Published:** 2024-11-09

**Authors:** Eva M. Malecore, Markus Fischer

**Affiliations:** ^1^ Botanical Garden of the University of Bern Bern Switzerland; ^2^ Institute of Plant Sciences University of Bern Bern Switzerland

**Keywords:** caucalidion, common species, phylogenetic distance, pollen recipient, self‐incompatible species

## Abstract

The mechanisms underlying plant species distribution and abundance have been long studied in ecology. However, the role of heterospecific pollen interference in shaping these patterns needs more attention. Species distribution and abundance are important factors determining whether a species is endangered or not; thus, understanding the impact of heterospecific pollen interference on rare species could help to inform conservation strategies aimed at preserving plant communities. In this study, we conducted a multispecies experiment using eight co‐occurring and co‐flowering plant species with varying rarity levels in Switzerland. We performed cross‐pollinations by hand between nearly all species pairs and measured seed set (whether a flower produces seed) and seed number (number of seeds per flower) as outcomes. We looked at the effects of rarity status, self‐compatibility, and recipient‐donor relatedness on heterospecific pollen interference. Contrary to expectations, neither seed set nor seed number were affected by heterospecific pollen deposition. Self‐compatible species had a higher seed set probability, but this was independent from species rarity. In our study setting, heterospecific pollen interference seems to have only minor effects on seed set and seed number, and consequently on recruitment. Thus, heterospecific pollen interference seems to play only a minor role in shaping plant species distribution and abundance. Nevertheless, the higher impact of heterospecific pollen deposition on rare and closely related species, as well as the importance of conspecific pollen loss, might need further investigation for both in situ and ex‐situ conservation strategies.

## Graphical abstract

In a multispecies greenhouse experiment, we performed hand pollination treatments between eight co‐occurring and co‐flowering plant species with varying rarity levels in Switzerland. We found that overall heterospecific pollen interference did not affect seed set nor seed number, used as proxies of recruitment, for neither common nor rare species. We further could show that heterospecific pollen tended to be higher for closely related and rare pollen recipient‐donor pairs, which needs further investigation.

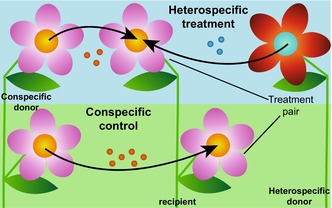

## 1 Introduction

Different aspects of the drivers of plant species distribution and abundance have been studied. Three main drivers are usually suggested to shape plant species distribution and abundance: abiotic factors, dispersal, and biotic interactions (Soberon 2007; Boulangeat, Gravel, and Thuiller 2012). Abiotic factors, such as soil moisture, temperature, and nutrient availability, influence species distribution and abundance in relation to a species' fundamental niche (Chase and Leibold 2003). Limited dispersal ability can prevent a species from reaching suitable habitats, even if these are available. Conversely, excellent dispersal ability can enable a species to colonize unsuitable sites through continuous immigration (Pulliam 2000). Biotic interactions, including both competition and facilitation among plant species, as well as interactions at other trophic levels such as predation, herbivory, and pollination, can also influence species distribution and abundance (Meier et al. 2010).

Pollinators can mediate indirect plant–plant interactions by acting as vectors, even between non‐neighboring individuals, due to their ability to move freely and cover long distances. While pollinators provide the essential function of pollination, they can also have negative effects. Recent studies have shown that, among other things, pollinators can transfer viruses between different species (Fetters 2023). More importantly, pollen mixes from different species can move between different donor and recipient species, a mechanism known as interspecific pollen transfer. Interspecific pollen transfer has two components: conspecific pollen loss and heterospecific pollen interference (HPI hereafter) (Morales and Traveset 2008; Ashman and Arceo‐Gómez 2013). Conspecific pollen loss refers to the reduction of pollen transferred between conspecific flowers due to loss to a heterospecific recipient. HPI refers to the reduction in reproductive output in the presence of heterospecific pollen (HP hereafter), despite the presence of conspecific pollen (CP hereafter) that could fertilize the ovules. HPI thus can potentially impact the female fitness of the recipient species through reduced seed set (Morales and Traveset 2008), while conspecific pollen loss can impact the male fitness of the donor species (Waser 1978) through reduced pollen transfer.

Previous studies have explored HPI between native and alien species (e.g., Suárez‐Mariño et al. 2019; Malecore et al. 2021). However, to the best of our knowledge, no study has specifically addressed the role of interspecific pollen transfer and, in particular, HPI between co‐occurring rare and common native species. Given that species distribution and abundance are crucial factors in determining a species' endangerment status, understanding the mechanisms of heterospecific pollen interference for rare species could provide insights for both in‐sit and ex‐situ conservation strategies aimed at preserving plant populations.

To mitigate HPI, plant species can either avoid or reduce heterospecific pollen deposition or evolve tolerance to it (Arceo‐Gómez, Raguso, and Geber 2016; Streher et al. 2020; Hao, Fang, and Huang 2023). Avoidance or reduction mechanisms can occur at the pre‐pollination stage through alterations in flower phenology, the development of flower restrictiveness, reliance on specialized pollinators, or the use of different deposition sites on the pollinator's body (Montgomery and Rathcke 2012). Tolerance mechanisms occur at the post‐pollination stage through pollen‐stigma or pollen‐pollen interactions. Tolerance is expected to evolve after exposure to heterospecific pollen. Therefore, in a plant community, if no avoidance or reduction mechanism prevents heterospecific pollen deposition, we can expect co‐flowering species sharing common pollinators to evolve mechanisms to tolerate HPI.

In a co‐flowering plant community, it is expected that overall common and abundant species receive more frequent visits from pollinators, while rare species receive fewer visits. Thus, according to the tolerance hypothesis (Hao, Fang, and Huang 2023), both rare and common species should be adapted to receive heterospecific pollen from other common species. On the other hand, both common and rare species should receive heterospecific pollen less frequently from other rare species. A reduced exposure means a lower need and chance to adapt to potential negative effects from heterospecific pollen. We predict that both common and rare species will experience HPI from rare donors but not from common donors.

The breeding system or self‐compatibility of donor and recipient species could be another factor determining the strength of HPI for co‐occurring species. Self‐incompatible species present either mechanical or chemical mechanisms to avoid self‐pollination (de Jong, Waser, and Klinkhamer 1993), and these mechanisms might similarly help in avoiding HPI. Thus, self‐incompatible species could be better equipped against HPI. In a conservation context, self‐compatibility could represent, in some cases, the only way for small populations to persist; thus a higher susceptibility to HPI for rare self‐compatible species could further endanger them.

Another factor that has received attention in relation to HPI is the recipient‐donor species relatedness. For example, due to similar recognition mechanisms, it could be that only pollen from closely related species germinate on the stigma of the recipient species. Therefore, HPI might be reduced among distantly related species. While in a previous study we showed that the phylogenetic distance between recipient and donor species did not affect the overall strength of HPI (Malecore et al. 2021), this pattern could change depending on the commonness or rarity of recipient and donor species.

In this study, we conducted hand‐pollination experiments on a total of eight co‐occurring and co‐flowering species, collected from wild populations in Switzerland. Five of these species are rare, and three are common in Switzerland. We will refer to species rarity or commonness as species status. We performed pairwise heterospecific pollen crosses as well as conspecific control treatments and measured seed set and seed number as our outcome variables. Seed set and seed number serve as proxies for reproductive success. We asked the following questions: (1) Does heterospecific pollen reduce seed set and seed number for common and rare recipient species, and does this reduction depend on recipient and donor status? (2) Does heterospecific pollen reduce seed set for self‐compatible and self‐incompatible recipient species, and does this reduction depend on recipient and donor self‐compatibility? (3) Does heterospecific pollen interference depend on recipient and donor relatedness? By addressing these questions, we aimed to shed new light on the complex interplay of factors that determine the distribution and abundance of plant species within co‐flowering communities. Ultimately, gaining a deeper understanding of the mechanisms underlying heterospecific pollen interference could help inform conservation efforts aimed at preserving endangered species.

## 2 Material and Methods

### 2.1 Study Species

**FIGURE 1:**
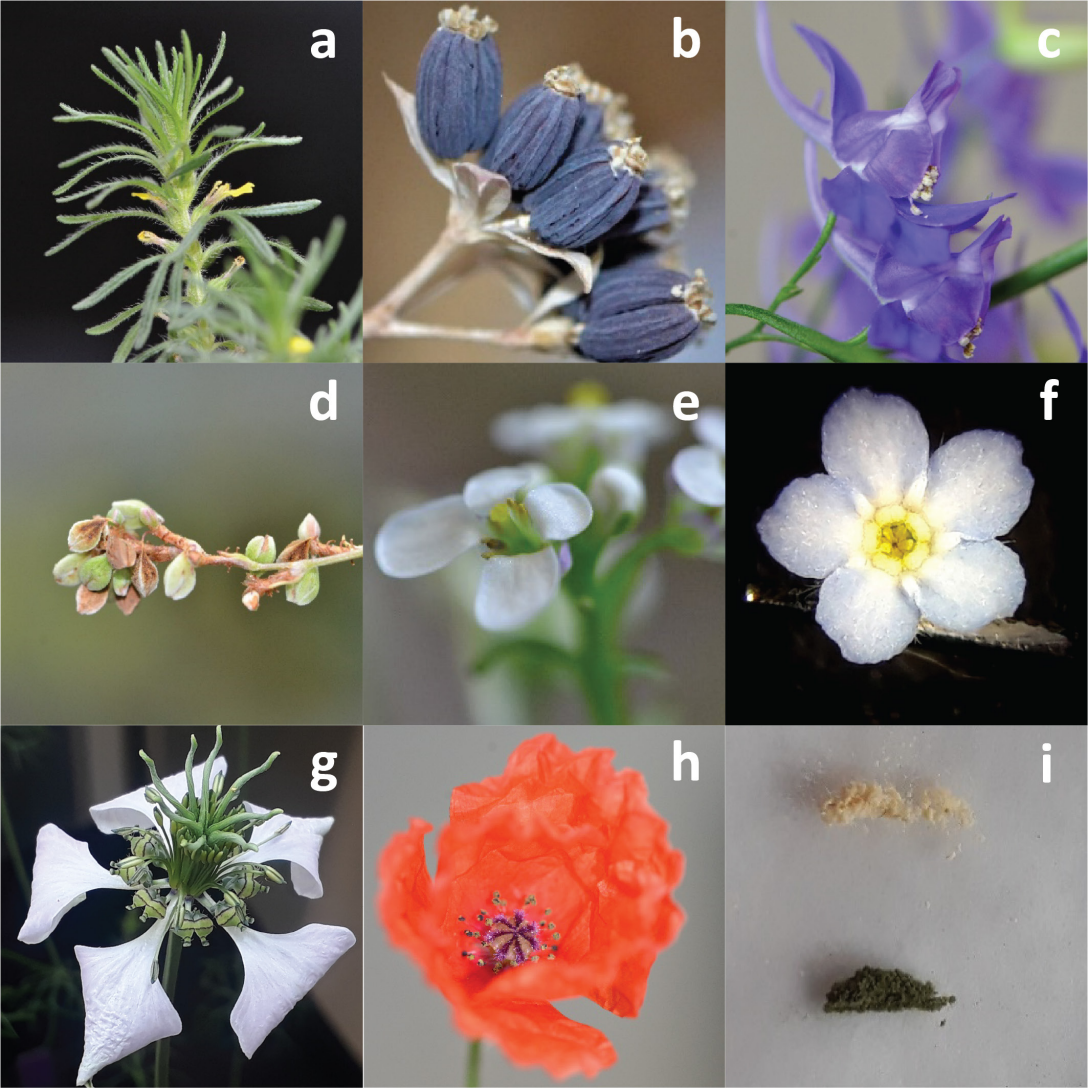
Depiction of the eight species used in the experiment (a) *Ajuga chamaepitys* (b) *Bupleurum rotundifolim* (c) *Consolida regalis* (d) *Fallopia convolvulus* (e) *Iberis amara* (f) *Myosotis arvensis* (g) *Nigella arvensis* (h) *Papaver rhoeas* and (i) an example of the pollen of two species (*Consolida regalis* on top and *Papaver rhoeas* on bottom) for heterospecific pollen treatment.

We used a total of eight species (see Figure 1; Table S1) with different distributions and IUCN red list status within Switzerland. All species are co‐occurring in the “arable vegetation of calcareous soils” habitat (“Caucalidion,” according to the classification by Delarze et al. 2015), insect‐pollinated, and have overlapping flowering times in nature (Landolt et al. 2010; Lauber, Wagner, and Gygax 2018). We classified species as “common” if their IUCN status in Switzerland was “least concern,” and as rare otherwise. IUCN status in Switzerland correlated strongly with the number of observations within Switzerland pooled between the years 2000 and 2020 (Info Flora database n.d, cor: 0.86, *p*‐value < 0.001). Breeding system (self‐compatible vs. self‐incompatible) was extracted from the BiolFlor database (Kuehn, Durka, and Klotz 2004). To test the relationship between recipient‐donor relatedness and heterospecific pollen interference, we constructed a phylogenetic tree for our species (see Figure S1) by pruning a modified version (Malecore et al. 2018) of the dated DaPhnE supertree of Central European plant species (Durka and Michalski 2012) and then calculated the phylogenetic distance using the cophenetic function of the “ape” package (Paradis, Claude, and Strimmer 2004) in R (R Core Team 2023).

### 2.2 Experimental Set‐Up

We sowed all species into 12 cm × 17 cm trays filled with “Seedling substrat” potting soil (Klasmann‐Deilmann GmbH, 49741 Geeste, Germany) and put them into the dark cold storage room at −4°C for stratification between 5 and 8 weeks. Once seeds started to germinate, we moved the trays to a greenhouse compartment. We transplanted seedlings into 11 cm × 11 cm × 12 cm pots filled with “Selmaterra” (fertilized heavy soil with 30% volume peat, see Table S2). We randomized pots on tables of a single pollinator‐free greenhouse compartment (14‐h light cycle, heating starting at 10°, cooling starting at 20°C) and watered as well as fertilized regularly. We treated aphids and fungi whenever necessary.

**FIGURE 2:**
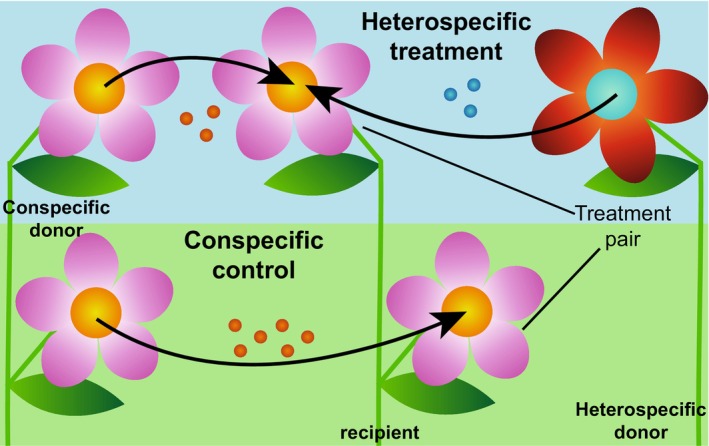
Graphic representation of the treatments used in the experiment. For the heterospecific treatment, a mix of conspecific pollen from a different individual and of heterospecific pollen from a heterospecific individual was applied to the recipient flower. For the conspecific control, on the same recipient plant a second flower was treated with conspecific pollen only. Both heterospecific and conspecific treatments of the same pair would use pollen from the same conspecific pollen donor individual. Pollen amount used was always saturating.

All species flowered between May 2021 and October 2021. To ensure continued flowering, we regularly deadheaded the plants. To assess the effect size of heterospecific pollen interference, we performed hand pollinations between and within nearly all species and measured seed set (yes/no) and seed number. Cross‐pollination between *Iberis amara* as recipient and *Bupleurum rotundifolium* as donor did not succeed and could not be repeated due to lack of flowers. For the heterospecific pollen treatment, we prepared a saturated mix of conspecific pollen and heterospecific pollen and applied it to the stigma of the recipient flower. For each flower treated with heterospecific pollen mixture, we treated a second flower on the same individual on the same day with conspecific pollen only as a control, using the same conspecific pollen donor that we used for the heterospecific pollen mix. Conspecific pollen and heterospecific pollen were from a single individual, respectively. The two flowers with heterospecific and conspecific treatment would constitute a pair with the same “pair ID” (see Figure 2). Pollen grain number per anther differed greatly depending on individual and on anther ripeness (personal observation from preliminary pollen counts with a Neubauer counting chamber) thus, we standardized treatment by always applying a pollen amount above saturation level. We extracted the pollen for the treatments from the anthers by tapping them on a glass slide and with the help of tweezers, and then mixed it for the heterospecific treatment. We then applied the pollen mixture (HP) or the conspecific pollen (CP) to the open stigma of the recipient flower using tweezers. To avoid selfing, we emasculated recipient flowers by removing the anthers some days before treatment. For some species (*Bupleurum rotundifolium*, *Fallopia convolvulus*, *Myosotis arvensis*), anther removal would cause too much flower damage due to the small size, thus anthers were not removed. For these species, selfing could not be completely excluded.

For each donor‐recipient combination, we treated between 2 and 16 flower pairs (HP and CP; median: 12 flower pairs per donor‐recipient combination; see Table S3). We collected seeds after ripening and counted them either by hand or by using an imaging method with imageJ (Abramoff, Magalhaes, and Ram 2004) (for *Papaver rhoeas*, see “Protocol seed counting in ImageJ” in Supporting Information).

### 2.3 Statistical Analysis

#### 2.3.1 Do Pollen Type and Recipient Status Affect Seed Set and Seed Number?

To test whether seed set and seed number are affected by pollen type (conspecific vs. heterospecific) and whether the effect size depends on recipient status (common vs. rare), and to account for the high proportion of zeroes (~24%), we ran a hurdle model using the function *glmmTMB* of the homonymous package (Brooks et al. 2017). In a hurdle model, zero counts and non‐zero counts are treated as two separate categories, meaning that a binomial model is fitted for zeroes versus non‐zeroes (the “zero‐inflated” model) and a separate model for the non‐zero counts only (“conditional model”). For the conditional model, we used a truncated negative binomial error distribution (“truncated nbinom2” in *glmmTMB*). We implemented the same formula for both the zero‐inflated and the conditional model, with pollen type (conspecific = 0, heterospecific = 1), recipient status (common = 0, rare = 1), and their interaction as fixed effects. To account for non‐independence, we included pair ID, treatment date, recipient species, recipient individual ID, and donor individual ID as random factors.

After running the models, we used the functions *emmeans* and *pairs* of the *emmeans* package (Lenth, 2023) to calculate the 95% confidence intervals of the estimated marginal mean for each group (conspecific and heterospecific treatments for common and rare recipients) and to test for significance of the comparisons of interest (conspecific vs. heterospecific treatment for common recipients, conspecific vs. heterospecific treatment for rare recipients).

#### 2.3.2 Does Donor Status Affect Seed Set and Seed Number?

To explore in more detail the effects of donor and recipient status on seed set and seed number, we separately analyzed a subset including only the HP treatment. We ran a hurdle model using the function *glmmTMB* of the homonymous package with recipient status (common = 0, rare = 1), a dummy factor indicating whether the heterospecific pollen donor was of the same or the opposite status (same = 0, opposite = 1), as well as their interaction as fixed effects. We included the same random factors as in the previous model (pair ID, treatment date, recipient species, recipient individual ID, and donor individual ID).

After running the models, we used the functions *emmeans* and *pairs* of the *emmeans* package to calculate the 95% confidence intervals of the estimated marginal mean for each group (heterospecific treatment for common and rare recipients with common and rare donors) and to test for significance of the comparisons of interest (heterospecific treatment: common donors vs. rare donors on common recipients, common donors vs. rare donors on rare recipients).

#### 2.3.3 Do Recipient and Donor Self‐Compatibility Affect Seed Set and Seed Number?

To test whether seed set and seed number are affected by the self‐compatibility of recipient and donor species in interaction with heterospecific pollen deposition, we repeated the same analyses as above, replacing recipient and donor status with recipient and donor self‐compatibility.

#### 2.3.4 Does Recipient‐Donor Relatedness Affect Seed Number?

To test whether the phylogenetic distance between recipients and donors affects seed set and seed number, we calculated for all non‐zero counts the log‐response ratio of seed number with HP treatment on seed number with CP treatment within each pair (same pair ID). We then fitted a Gaussian *glmmTMB* model including recipient‐donor phylogenetic distance, recipient status (common = 0, rare = 1), a dummy factor indicating whether the heterospecific pollen donor was of the same or the opposite status (same = 0, common = 1), as well as all their interactions as fixed effects. To account for non‐independence, we included treatment date, recipient species, donor species, recipient individual ID, and donor individual ID as random factors.

After running the model, we used the functions *emmtrends* of the *emmeans* package to calculate the estimated trends with their 95% confidence intervals for the relationship between log‐response ratio and recipient‐donor relatedness for each group (common recipient with common donor, common recipient with rare donor, rare recipient with rare donor, rare recipient with common donor).

For all models, we inspected Pearson residuals for homogeneity of variance against all grouping variables.

## 3 Results

At the end of the experiment, we successfully treated 1320 flowers (660 flower pairs). Zero counts made up 24% of the total data, and seed number ranged from 1 to 1282 seeds per flower (depending on species), with a median of four seeds per flower.

### 3.1 The Effect of Pollen Type, Recipient and Donor Status on Seed Set and Seed Number

Heterospecific pollen reduced neither seed set nor seed number (see Figures S2 and S3; Tables S4 and S5). Overall, rare species tended to have a higher seed number compared to common species, but this trend was not significant. Similarly, recipient status and donor status affected neither seed set nor seed number (see Figures S4 and S5; Tables S6 and S7).

### 3.2 The Effect of Recipient and Donor Self‐Compatibility on Seed Set and Seed Number

**FIGURE 3:**
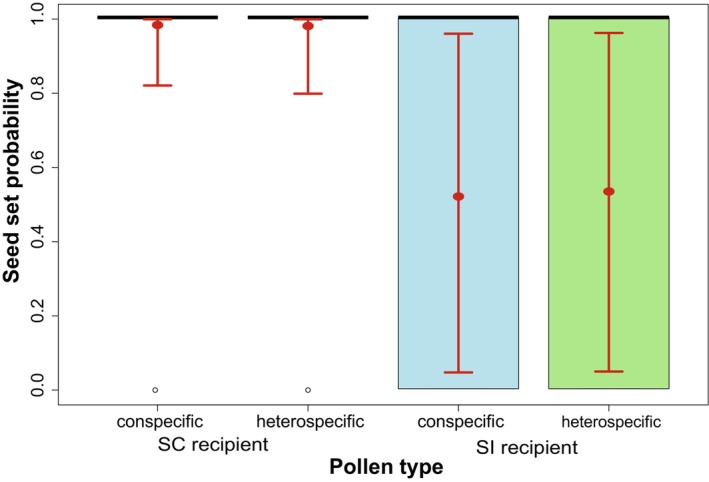
Seed set probability for conspecific and heterospecific treatments for self‐compatible (SC) and self‐incompatible (SI) recipient species (light blue: Conspecific treatment; light green: Heterospecific treatment). The estimated marginal means with their 95% confidence intervals for each group are plotted in red on top of the boxplots of the raw data. Overall, SC species have a higher seed set probability compared to SI species, independently of pollen treatment (SC/SI odds ratio = 0.02, SE = 0.04, *p*‐value = 0.05).

Self‐incompatible recipient species showed a lower seed set probability compared to self‐compatible species (SI/SC odds ratio = 0.0206, SE = 0.0416, *p*‐value = 0.05). This effect was independent of pollen‐type treatment (see Figure 3 and Tables 1 and 2). Seed number was not affected by the self‐compatibility of recipient or donor species (see Figures S6 and S8; Tables S8 and S9).

**TABLE 1:** Hurdle model for seed set and seed number for the full dataset including pollen type and self‐compatibility of recipient species as predictor variables (SI: Self‐incompatible).

Response variable (*n* = 1320)	Seed set (yes/no)		Seed number	
Parameter	Estimate (SE)	*p* (>|*z*|)	Estimate (SE)	*p* (>|*z*|)
Intercept (Type CP)	−4.068 (1.301)	0.002	0.6689 (1.363)	0.624
Type HP	0.1479 (0.271)	0.585	0.0003 (0.061)	0.996
Recipient SI	3.9826 (2.031)	0.050	1.4223 (1.422)	0.516
Type HP:Recipient SI	−0.2018 (0.413)	0.625	−0.1416 (−0.142)	0.151

Random terms	SD	SD
Pair ID (660)	6.204 × 10^−10^	3.278 × 10^−11^
Recipient species (8)	6.870	8.648
Treatment date (70)	2.202	6.951 × 10^−1^
Recipient individual ID (199)	−3.342	1.325 × 10^−1^
Donor individual ID (328)	3.654204 × 10^−10^	1.291 × 10^−9^
*AIC:*	7631.2	

*Note:* The “zero‐inflated” model is binomial with a logit link function, while the “conditional model” for the non‐zero counts is a truncated negative binomial model with a log link function (“nbinom2” in *glmmTMB*). The variance for the binomial model is given by *np(1 − p)* where *n* is the number of trials and *p* the probability of a zero, while the variance for the negative binomial model is given by *mean(1 − ɸ)*, where the dispersion parameter *ɸ* allows for a larger variance. By default, the zero‐inflation model coefficients give the likelihood of a zero.

**TABLE 2:** Back‐transformed estimated marginal means and confidence intervals for each grouping for the first hurdle model (full dataset) including pollen type (CP: Conspecific, HP: Heterospecific) and self‐compatibility of recipient species as predictor variables (SC: Self‐compatible, SI: Self‐incompatible).

Group		Seed set			Seed number		
Recipient	Donor	Probability (SE)	Lower 95% CI	Upper 95% CI	Mean (SE)	Lower 95% CI	Upper 95% CI
SC	CP	0.98 (0.02)	0.82	1.00	1.95 (2.66)	0.14	28.22
	HP	0.98 (0.02)	0.8	1.00	1.95 (2.66)	0.14	28.23
SI	CP	0.52 (0.39)	0.05	0.96	8.1 (13.98)	0.27	239.17
	HP	0.53 (0.39)	0.05	0.96	7.03 (12.14)	0.24	207.69

*Note:* For an easier interpretation, for the binomial model the probability of success is reported (by default, in a hurdle model, the probability of failure is estimated).

### 3.3 The Effect of Recipient‐Donor Relatedness on Seed Number

Overall, phylogenetic relatedness between recipient and donor species did not affect the strength of HPI. For rare recipients with rare donors, HPI tended to decrease with relatedness (Δ_lrr_/Δ_PD_ = 0.0017, SE = 0.0010, *p*‐value = 0.08), with more distantly related recipient‐donor species pairs less affected by HP. (see Figure S9 and Tables 3 and 4).

**TABLE 3:** Gaussian model with the log‐response ratio of seed number with HP treatment to seed number with CP treatment within flower pairs (same pair ID) for non‐zero counts as response variable, and recipient‐donor phylogenetic distance as well as recipient and donor status as predictor variables.

Response variable (*n* = 453)		
Parameter	Estimate (SE)	*p* (>|*z*|)
Intercept (Recipient common)	0.2124 (0.210)	0.860
Recipient rare	−0.6399 (0.132)	0.604
Opposite status donor	0.0970 (0.1333)	0.942
PD	−0.0008 (0.005)	0.866
Recipient rare: Opposite status donor	−0.0074 (0.500)	0.996
Recipient rare: PD	0.0025 (0.005)	0.608
Opposite status donor: PD	−0.0010 (0.005)	0.860
Recipient rare: Opposite status donor:PD	−0.0010 (0.006)	0.897

Random terms	SD
Recipient species (8)	2.651 × 10^−5^
Donor species (8)	1.076 × 10^−5^
Treatment date (55)	1.745 × 10^−1^
Recipient individual ID (159)	2.877 × 10^−1^
Donor individual ID (250)	1.600 × 10^−1^
Residual	7.099 × 10^−1^
*Dispersion estimate*	0.504

**TABLE 4:** Estimated marginal trend with standard error and 95% confidence interval for the slope of the relationship between log‐response ratio and recipient‐donor phylogenetic distance, for each recipient‐donor status combination.

Recipient	Donor	Δ_lrr_/Δ_PD_ (SE)	Lower 95% CI	Upper 95% CI
Common	Common	−0.0008 (0.0048)	−0.0102	0.0085
	Rare	−0.0017 (0.0027)	−0.007	0.0035
Rare	Rare	0.0017 (0.001)	−0.0002	0.0036
	Common	0.0015 (0.0025)	−0.0034	0.0065

## 4 Discussion

In our study, heterospecific pollen interference did not affect seed set or seed number, i.e., it did not affect whether or not a flower would produce at least one seed or the amount of seeds produced. Rare species had a tendency to produce more seeds, but this trend was not significant. Breeding systems did affect seed set, but not in relation to conspecific or heterospecific pollen treatments, with self‐incompatible species less likely to set seed compared to self‐compatible species. Lastly, for rare recipients treated with pollen from rare donors, more distantly related recipient‐donor species pairs tended to have a lower reduction in seed number compared to closely related recipient‐donor species pairs. Hereafter we discuss these results as well as potential ecological and evolutionary implications.

In a co‐flowering community, we can expect common species to receive pollen from a rare species infrequently; thus, an adaptation to that type of pollen is unlikely. On the other hand, a rare species is likely to receive frequently pollen from common species, thus making an adaptation to heterospecific pollen receipt more likely, as predicted by the tolerance hypothesis (Hao, Fang, and Huang 2023). Indeed, in a study by Arceo‐Gómez, Raguso, and Geber (2016), the authors showed how HP tolerance for a *Clarkia* species did depend on previous exposure of the population to HP, but rather than acting on the recipient individual, would act on the donor individual by improving CP performance. On the other hand, such adaptation was not observed for a congeneric *Clarkia* species, suggesting that adaptation is context‐ and species‐specific. Adaptation could explain the low effect of HP overall in our study species. On the other hand, even though our study species do co‐occur and co‐flower in nature, the seed material did not consistently originate from populations co‐occurring at the local scale, but co‐occurring only at the regional scale, thus missing potential adaptations at the population level. Further, the co‐occurrence history of the populations from which the seed material was collected is unknown.

Another factor we analyzed is the evolutionary relatedness, measured as phylogenetic distance, between recipient and donor species in interaction with recipient and donor status. While overall no pattern emerged, we could show a trend toward a decrease of HPI for more distantly related recipient‐donor pairs when both were rare. The likely reason for the absence of these patterns for common recipients and rare recipients with common donors could be the lack of close relatives for these groups in our study species set. Indeed, the range for these groups included only phylogenetic distances larger than 189 × 10^6^ years. For closely related recipient‐donor pairs, a stronger HPI could be caused by similar recognition systems between recipient stigma and donor pollen grains (Moyle and Nakazato 2010; Moyle, Olson, and Tiffin 2004; Scopece, Widmer, and Cozzolino 2008). For a better understanding of these patterns, a study species set with a broader range should be used.

In this study we looked only at pairwise HP interactions, while in a plant community it is likely to have multi‐species mixes of HP that are transferred between flowers. Ashman and Arceo‐Gómez (2011) performed HP hand‐pollinations with mixes up to three species and showed that HPI increased with the number of heterospecific pollen donors. Further, the strength depended on specific species composition. Some species are known to produce strongly allelopathic pollen (Kanchan and Chanra 1980), but in our study it did not look like a specific species had a consistent negative effect on all other species in terms of seed number (Figure S10).

For our species, self‐incompatible species showed an overall lower seed set, while the breeding system did not affect seed number or HPI in any way. Self‐compatibility might play a role, especially in a natural community where HP can act through the mentor effect (de Nettancourt 1997) and allow for self‐fertilization even in self‐incompatible flowers, with consequent ovule abortion (Lynn, Sullivan, and Galen 2022). In our study, we showed how self‐incompatible species are less likely to produce seeds even when enough pollen is present. We emasculated our recipient species whenever possible prior to treatment, but due to the small flower size, three out of eight species were left with their anthers to avoid complete flower abortion. These three species (*Bupleurum rotundifolium*, *Fallopia convolvulus,* and *Myosotis arvensis*) are all self‐compatible. Thus, one explanation could be that seed set induced by selfing is more secure compared to seed set from outcrossed pollen, despite the genetic advantages of outbreeding (Goodwillie and Weber 2018).

Specific flower morphology and in particular a smaller stigma size in restrictive flowers (i.e., flowers with a reduced access to the flower interior) have been shown to reduce HP deposition while at the same time increasing CP deposition (Montgomery and Rathcke 2012). In our study, we did not analyze the effect of flower morphology or flower traits, since due to our small sample size in species number (eight species in total), species and trait would be confounded. In a natural community, flower morphology would also play an important role in terms of pollinator sharing and flower constancy (the tendency of pollinators to forage on the same flower type, Waser 1986), since some flowers are adapted to specific groups of pollinators, and thus sharing among these species is more likely. For example, both *Ajuga chamaepitys* and *Fallopia convolvulus*, being lip flowers, rely on bumblebees as their most common pollinators (Kuehn, Durka, and Klotz 2004) (see Table S1 for a complete list of typical pollinators for each species). Morphological differences and pollinator type can also drive the direction (e.g., from species A to species B but not from species B to species A) and amount of pollen transferred (Fang and Huang 2013). In our study, we used all species both as donors and as recipients and used a saturated amount of pollen, but this could differ in natural conditions, where pollen limitation is common (Burd 1994; Knight et al. 2005; Larson and Barrett 2000).

While interspecific pollen transfer seems to be widespread, the magnitude and the effect on reproductive output are highly context dependent. In line with our findings, several studies showed limited or no effect of HP deposition on seed production (see Morales and Traveset 2008 and references within), while conspecific pollen loss seems to be the more important component of interspecific pollen transfer (Mitchell et al. 2009). Further, the impact of HP deposition has been shown to depend on timing of HP application, with seed set more affected when heterospecific pollen is applied prior to conspecific pollen, compared to a simultaneous mixed application (see Morales and Traveset 2008 and references within) as in this study.

While in our study we did not find any strong effect of HP on seed set and seed number, HPI remains an important aspect of co‐flowering communities (Ashman and Arceo‐Gómez 2013), since it allows species to affect other species without direct competition and at a distance above the direct interactions. In our study we could do a full recipient‐donor combination treatment for a total of eight species, which could mean a reduced statistical power when comparing groups within. While using a multi‐species approach can be labour‐intensive, we suggest to increasing the number of species for further studies or performing multiple experiments to join the data in a meta‐analysis in order to increase statistical power. In a co‐flowering community, we can expect a variable and complex pollination landscape that could promote the evolution of flower morphology and avoidance mechanisms also due to HPI. At the same time, the lack of stable conditions and the variability of the direction of selection between years and generations (Feinsinger 1987) could hamper adaptation to HP deposition, preventing the emergence of clear patterns. It seems that while mechanisms such as HPI and adaptations to it play a role in shaping plant communities, these patterns are highly variable depending on the context and on the species observed. We conclude that heterospecific pollen interference plays a minor role for rare plant species in our study system. Rather, other factors, like pollen limitation mediated by low pollinator visitation rates or conspecific pollen loss to heterospecific recipient flowers, are likely to affect rare plant species. Adaptation and species‐specific interactions may explain the low overall effect of HPI in our study. The complexity of multi‐species interactions and the specific composition of heterospecific pollen mixes may further influence the strength of HPI. Additional research is needed to explore these factors and their implications for both in‐sit and ex‐situ conservation strategies.

## Author Contributions

**Eva M. Malecore:** conceptualization (lead), data curation (lead), formal analysis (lead), investigation (lead), methodology (lead), project administration (lead), validation (lead), visualization (lead), writing – original draft (lead), writing – review and editing (lead). **Markus Fischer:** funding acquisition (lead), supervision (lead), writing – review and editing (supporting).

## Conflicts of Interest

The authors declare no conflicts of interest.

## Supporting information


Appendix S1.

## Data Availability

The data that supports the findings of this study will be available on Dryad doi:10.5061/dryad.x69p8czs3.
