# Supplementary material for: Pollen Interference Between Rare and Common Species
Source: Ecol Evol. 2024 Nov 9;14(11):e70505. doi: 10.1002/ece3.70505 (PMC11549573; doi:10.1002/ece3.70505)
Supplement: Supplementary file 1 — Appendix S1. [file ECE3-14-e70505-s001.docx]

# Supporting Information

## SI Tables

Table S 1 Plant species used for the hand-pollination experiment. All species are part of the habitat type Caucalidion (arable vegetation of calcareous soils). Seed material was collected from wild population and the region of origin as well as the collection date are reported. Rare species in light blue, common species in dark blue. Information on “Habitat” and “Flowering time (month)” was extracted from Flora Helvetica (Lauber, Wagner, & Gygax, 2018); on “IUCN Status in CH (2016)” from the Info Flora database; on“Self-compatibility” and “Typical pollinators” from the BiolFlor database (Kuehn, Durka, and Klotz 2004).

| **Species** | **Family** | **Habitat** | **Origin seed material** | **Collection date** | **IUCN Status in CH (2016)** | **Flowering time (month)** | **Selfcompatibility** | **Typical.pollinators** |
| --- | --- | --- | --- | --- | --- | --- | --- | --- |
| *Ajuga chamaepitys* | Lamiaceae | Caucalidion | Brig, Biela (VS) | September 2020 | VU – rare | 5-9 | self-compatible | bumblebees |
| *Bupleurum rotundifolium* | Apiaceac | Caucalidion | Montana (VS) | November 2020 | EN – rare | 5-8 | self-compatibel | beetles, flies, syrphids, wasps, medium tongued bees |
| *Consolida regalis* | Ranunculaceae | Caucalidion | Brig, Biela (VS) | September 2020 | VU – rare | 6-9 | self-incompatibel | bumblebees |
| *Fallopia convolvulus* | Polygonaceae | Caucalidion | Schmitten (GR) | September 2020 | EN – rare | 7-9 | self-compatibel | syrphids, bees |
| *Iberis amara* | Brassicaceae | Caucalidion | Bözingen (BE) | September 2020 | LC – common | 6-10 | self-incompatibel | bees, bumblebees, wasps, bombylides, syrphids |
| *Myosotis arvensis* | Boraginaceae | Caucalidion | Düdingen (FR) | October 2020 | LC - common | 4-10 | self-compatibel | bees, bumblebees, wasps, bombylides, syrphids |
| *Nigella arvensis* | Ranunculaceae | Caucalidion | Turtmann (VS) | September 2020 | CR - rare | 6-9 | self-compatibel | hymenopteres |
| *Papaver rhoeas* | Papaveraceae | Caucalidion | Wylerbad (BE) | July 2019 | LC - common | 5-9 | self-incompatibel | short tongued bees, syrphids, flies, beetles |

Table S 2 Soil types used for seedling germination and potting of the species used in the study.

| **Soil** | **Use** | **Composition (% volume)** | **Company** |
| --- | --- | --- | --- |
| Seedlingsubstrat potting soil | Stratification and seedling germination | - ca. 66% white sod peat, ca. 17% coconut pulp, ca 17% frozen black peat. - pH ca. 5.5; salt-content 0.7 mS. | Klasmann-Deilmann GmbH,  49741 Geeste,  Germany  +49 59 373 10 |
| Selmaterra | Potting | - 30% peat, 25% wooden fiber, 25% agricultural field soil, 20% bark and garden compost. - pH: ca. 6.8-7.2; salt-content ca. 1.8 mS. | Bigler Samen AG  Maienstrasse 8  CH-3602 Thoune, Switzerland  +41 33 227 57 36  Info@biglersamen.ch |

Table S 3 Number of combinations for each recipient-donor pair.

| **Recipient**  **Donor** | Ajuga chamaepitys | Bupleurum rotundifolium | Consolida regalis | Fallopia convolvulus | Iberis amara | Myosotis arvensis | Nigella arvensis | Papaver rhoeas |
| --- | --- | --- | --- | --- | --- | --- | --- | --- |
| Ajuga chamaepitys | 14 | 9 | 12 | 11 | 8 | 16 | 13 | 14 |
| Bupleurum rotundifolium | 2 | 7 | 14 | 11 | NA | 4 | 5 | 15 |
| Consolida regalis | 16 | 11 | 11 | 11 | 11 | 15 | 12 | 15 |
| Fallopia convolvulus | 14 | 10 | 12 | 11 | 7 | 15 | 13 | 16 |
| Iberis amara | 15 | 5 | 13 | 12 | 7 | 15 | 11 | 16 |
| Myosotis arvensis | 16 | 10 | 8 | 15 | 6 | 16 | 12 | 14 |
| Nigella arvensis | 14 | 10 | 10 | 10 | 9 | 15 | 14 | 15 |
| Papaver rhoeas | 16 | 12 | 14 | 14 | 11 | 16 | 14 | 15 |

Table S 4 Hurdle model for seed set and seed number for the full dataset including pollen type (CP: conspecific, HP: heterospecific) and recipient status as predictor variables. The “zero-inflated” model is binomial with a logit link function, while the “conditional model” for the non-zero counts is a truncated negative binomial model with a log link function (“nbinom2” in *glmmTMB*). The variance for the binomial model is given by *np(1-p)*where *n* is the number of trials and *p* the probability of a zero, while the variance for the negative binomial model is given by *mean(1- ɸ)*, where the dispersion parameter *ɸ*allows for a larger variance. By default, the zero-inflation model coefficients give the likelihood of a zero.

| **Response variable (n=1320)** | **Seed set (yes/no)** | | **Seed number** | |
| --- | --- | --- | --- | --- |
| **Parameter** | **Estimate (SE)** | **p-value (>\|z\|)** | **Estimate (SE)** | **p-value (>\|z\|)** |
| Intercept (Type CP) | -2.3632 (1.888) | 0.211 | 0.2562 (1.806) | 0.887 |
| Type HP | 0.0260 (0.265) | 0.922 | -0.1463 (0.079) | 0.063 |
| Recipient rare | -0.3202 (2.401) | 0.894 | 1.4911 (2.239) | 0.505 |
| Type HP:Recipient rare | 0.0869 (0.415) | 0.834 | 0.1468 (0.099) | 0.139 |
| **Random terms** | **SD** | | **SD** | |
| Pair ID (660) | 9.731 * 10^-5^ | | 1.484 * 10^-4^ | |
| Recipient species (8) | 3.188 | | 2.952 | |
| Treatment date (70) | 1.481 | | 8.360 * 10^-1^ | |
| Recipient individual ID (199) | 1.817 | | 3.652 * 10^-1^ | |
| Donor individual ID (328) | 6.132* 10^-6^ | | 8.282 * 10^-6^ | |
|  |  |  | **Dispersion parameter** $\boldsymbol{\varphi}$**:** 2.81 | |
| **AIC:** | 7634.4 | | | |

Table S 5 Back transformed estimated marginal means and confidence intervals for the first hurdle model (full dataset) including pollen type (CP: conspecific, HP: heterospecific) and recipient status as predictor variables. For an easier interpretation, for the binomial model the probability of success is reported (by default, in a hurdle model, the probability of failure is estimated).

| **Group** | | **Seed set** | | | **Seed number** | | |
| --- | --- | --- | --- | --- | --- | --- | --- |
| **Recipient** | **Type** | **probability (SE)** | **lower 95% CI** | **upper 95% CI** | **mean (SE)** | **lower 95% CI** | **upper 95% CI** |
| **Common** | **CP** | 0.91 (0.14) | 0.21 | 1.00 | 1.26 (2.33) | 0.04 | 44.48 |
|  | **HP** | 0.91 (0.15) | 0.20 | 1.00 | 1.1 (2.06) | 0.03 | 38.43 |
| **Rare** | **CP** | 0.94 (0.09) | 0.42 | 1.00 | 5.7 (7.68) | 0.42 | 79.02 |
|  | **HP** | 0.93 (0.10) | 0.40 | 1.00 | 5.7 (7.68) | 0.42 | 79.06 |

Table S 6 Hurdle model for seed set and seed number for heterospecific treatment only including recipient status and donor status as predictor variables. The “zero-inflated” model is binomial with a logit link function, while the “conditional model” for the non-zero counts is a truncated negative binomial model with a log link function (“nbinom2” in *glmmTMB*). The variance for the binomial model is given by *np(1-p)*where *n*is the number of trials and *p*the probability of a zero, while the variance for the negative binomial model is given by *mean(1- ɸ)*, where the dispersion parameter *ɸ*allows for a larger variance. By default, the zero-inflation model coefficients give the likelihood of a zero.

| **Response variable (n=660)** | **Seed set (yes/no)** | | **Seed number** | |
| --- | --- | --- | --- | --- |
| **Parameter** | **Estimate (SE)** | **p-value (>\|z\|)** | **Estimate (SE)** | **p-value (>\|z\|)** |
| Intercept (Recipient common) | -2.0934 (1.826) | 0.251 | 0.5568 (1.735) | 0.748 |
| Recipient rare | -0.5212 (2.343) | 0.824 | 1.1615 (2.252) | 0.589 |
| Opposite satus donor | 0.2877 (0.430) | 0.504 | -0.1638 (0.154) | 0.287 |
| Recipient rare: opposite status donor | 0.2099 (0.604) | 0.728 | 0.2444 (0.184) | 0.183 |
| **Random terms** | **SD** | | **SD** | |
| Pair ID (660) | 5.554 * 10^-5^ | | 1.068 * 10^-5^ | |
| Recipient species (8) | 3.048 | | 2.827 | |
| Treatment date (69) | 1.277 | | 7.277 * 10^-1^ | |
| Recipient individual ID (199) | 1.307 | | 4.945 * 10^-5^ | |
| Donor individual ID (327) | 5.050 * 10^-5^ | | 1.652 * 10^-5^ | |
|  |  |  | **Dispersion parameter** $\boldsymbol{\varphi}$**:** 2.06 | |
| **AIC:** | 3957.8 | | | |

Table S 7 Back transformed estimated marginal means and confidence intervals for each grouping fpr the second hurdle model (heterospecific treatment only) including recipient status and donor status as predictor variables. For an easier interpretation, for the binomial model the probability of success is reported (by default, in a hurdle model, the probability of failure is estimated)

| **Group (HP only)** | | **Seed set** | | | **Seed number** | | |
| --- | --- | --- | --- | --- | --- | --- | --- |
| **Recipient** | **Donor** | **probability (SE)** | **lower 95% CI** | **upper 95% CI** | **mean (SE)** | **lower 95% CI** | **upper 95% CI** |
| **Common** | **Common** | 0.89 (0.18) | 0.18 | 1.00 | 1.75 (3.03) | 0.06 | 52.33 |
|  | **Rare** | 0.86 (0.23) | 0.15 | 1.00 | 1.481 (2.57) | 0.05 | 44.29 |
| **Rare** | **Rare** | 0.93 (0.10) | 0.41 | 1.00 | 5.57 (7.19) | 0.44 | 69.88 |
|  | **Common** | 0.89 (0.14) | 0.30 | 0.99 | 6.04 (7.80) | 0.48 | 75.89 |

Table S 8 Hurdle model for seed set and seed number for heterospecific treatment only including self-compatibility of recipient and donor species as predictor variables (SC: self-compatible; SI: self-incompatible). The “zero-inflated” model is binomial with a logit link function, while the “conditional model” for the non-zero counts is a truncated negative binomial model with a log link function (“nbinom2” in *glmmTMB*). The variance for the binomial model is given by *np(1-p)*where *n*is the number of trials and *p*the probability of a zero, while the variance for the negative binomial model is given by *mean(1- ɸ)*, where the dispersion parameter *ɸ*allows for a larger variance. By default, the zero-inflation model coefficients give the likelihood of a zero.

| **Response variable (n=660)** | **Seed set (yes/no)** | | **Seed number** | |
| --- | --- | --- | --- | --- |
| **Parameter** | **Estimate (SE)** | **p-value (>\|z\|)** | **Estimate (SE)** | **p-value (>\|z\|)** |
| Intercept (Recipient SC) | -3.2133 (1.326) | 0.015 | 0.7879 (1.287) | 0.540 |
| Recipient SI | 3.1750 (2.054) | 0.122 | 1.4194 (2.082) | 0.495 |
| Opposite self-compatibility donor | -0.6520 (0.404) | 0.107 | -0.0714 (0.107) | 0.503 |
| Recipient rare: opposite self-compatibility donor | 0.7151 (0.682) | 0.294 | 0.0166 (0.192) | 0.931 |
| **Random terms** | **SD** | | **SD** | |
| Pair ID (660) | 1.001 * 10^-8^ | | 1.149 * 10^-10^ | |
| Recipient species (8) | 6.707 | | 7.668 | |
| Treatment date (69) | 1.872 | | 4.954 * 10^-1^ | |
| Recipient individual ID (199) | 1.698 | | 2.684 * 10^-9^ | |
| Donor individual ID (327) | 58.765 * 10^-9^ | | 3.154 * 10^-10^ | |
|  |  |  | **Dispersion parameter** $\boldsymbol{\varphi}$**:** 2.02 | |
| **AIC:** | 3955.2 | | | |

Table S 9 Back transformed estimated marginal means and confidence intervals for each grouping for the second hurdle model (heterospecific treatment only) including self-compatibility of recipient and donor species as predictor variable. For an easier interpretation, for the binomial model the probability of success is reported (by default, in a hurdle model, the probability of failure is estimated)

| **Group (HP only)** | | **Seed set** | | | **Seed number** | | |
| --- | --- | --- | --- | --- | --- | --- | --- |
| **Recipient** | **Donor** | **probability (SE)** | **lower 95% CI** | **upper 95% CI** | **Mean (SE)** | **lower 95% CI** | **upper 95% CI** |
| **SC** | **SC** | 0.96 (0.05) | 0.65 | 1.00 | 2.2 (2.83) | 0.18 | 27.37 |
|  | **SI** | 0.98 (0.03) | 0.77 | 1.00 | 2.05 (2.63) | 0.16 | 25.49 |
| **SI** | **SI** | 0.51 (0.40) | 0.04 | 0.96 | 9.09 (15.00) | 0.36 | 230.55 |
|  | **SC** | 0.49 (0.39) | 0.04 | 0.96 | 8.61 (14.17) | 0.34 | 216.67 |

## SI Figures

**
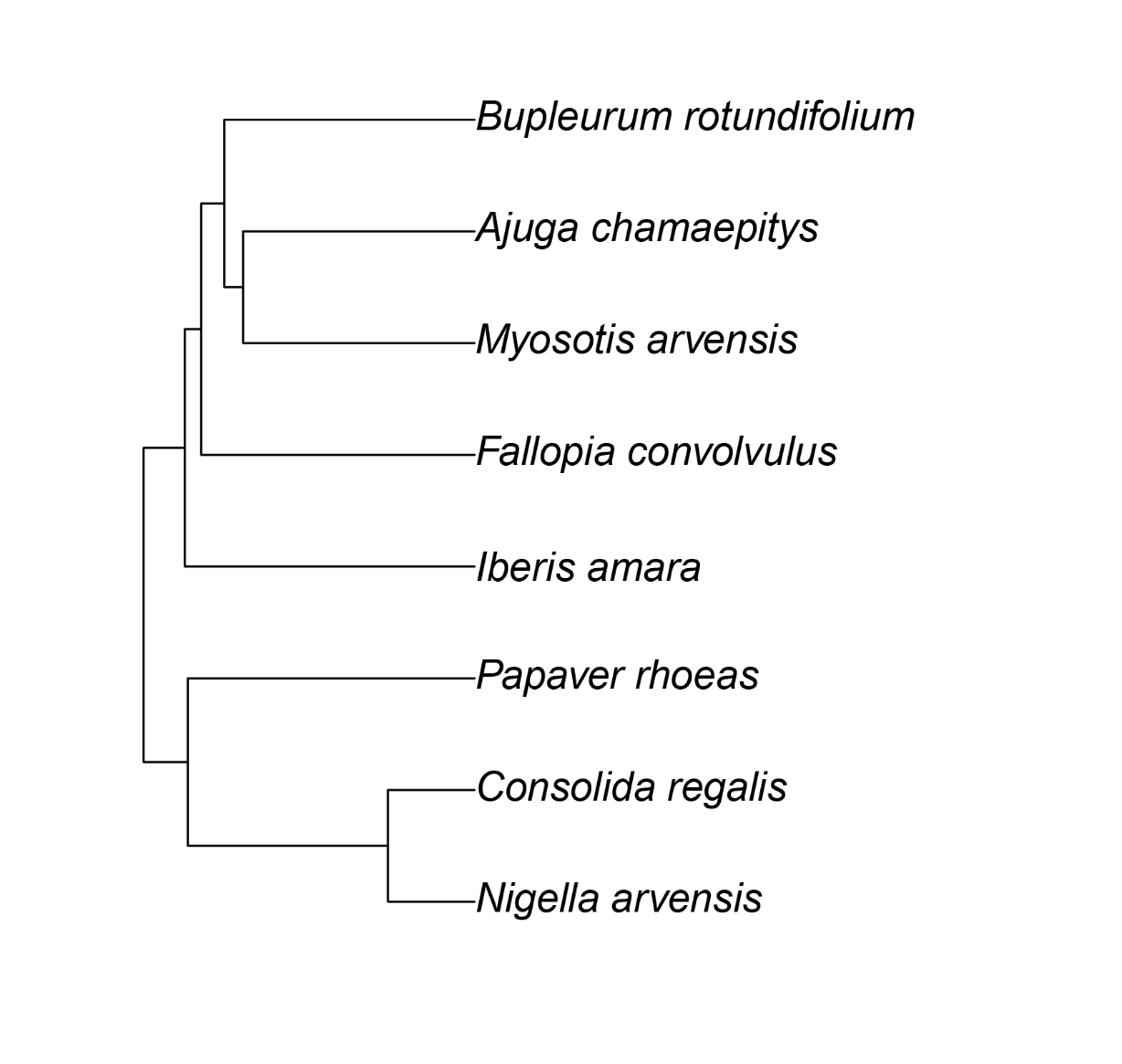
**

Figure S 1 Phylogenetic tree of species used in the study. The tree was obtained by pruning the dated DaPhnE supertree of Central European plant species (Durka & Michalski, 2012).


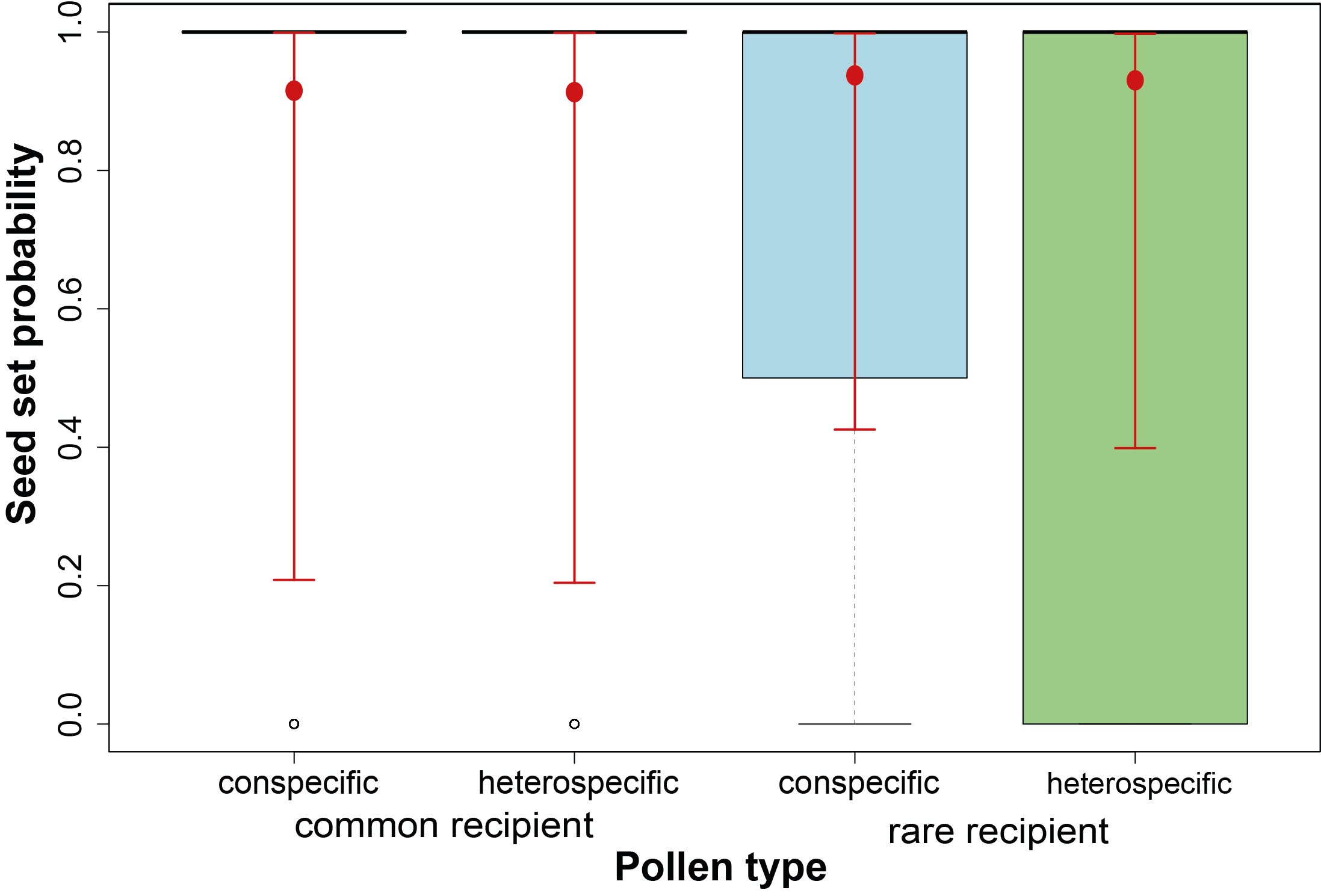


Figure S 2 Seed set probability for conspecific and heterospecific treatments for common and rare recipients (light blue: conspecific treatment; light green: heterospecific treatment). The estimated marginal means with their 95% confidence intervals for each group are plotted in red on top of the boxplots of the raw data.


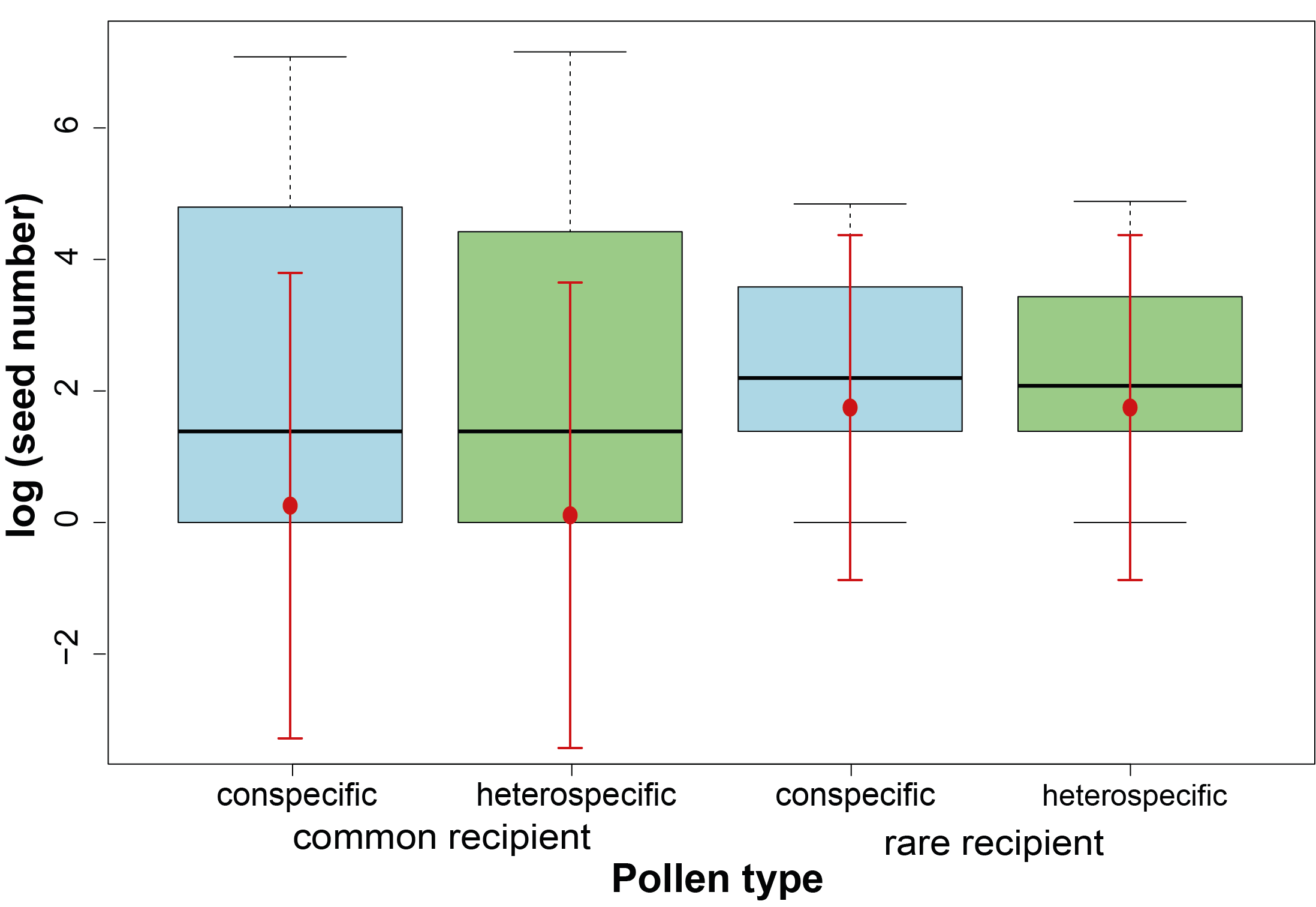


Figure S 3 Logged seed number for conspecific and heterospecific treatments for common and rare recipients (light blue: conspecific treatment; light green: heterospecific treatment). The estimated marginal means with their 95% confidence intervals for each group are plotted in red on top of the boxplots of the raw data.


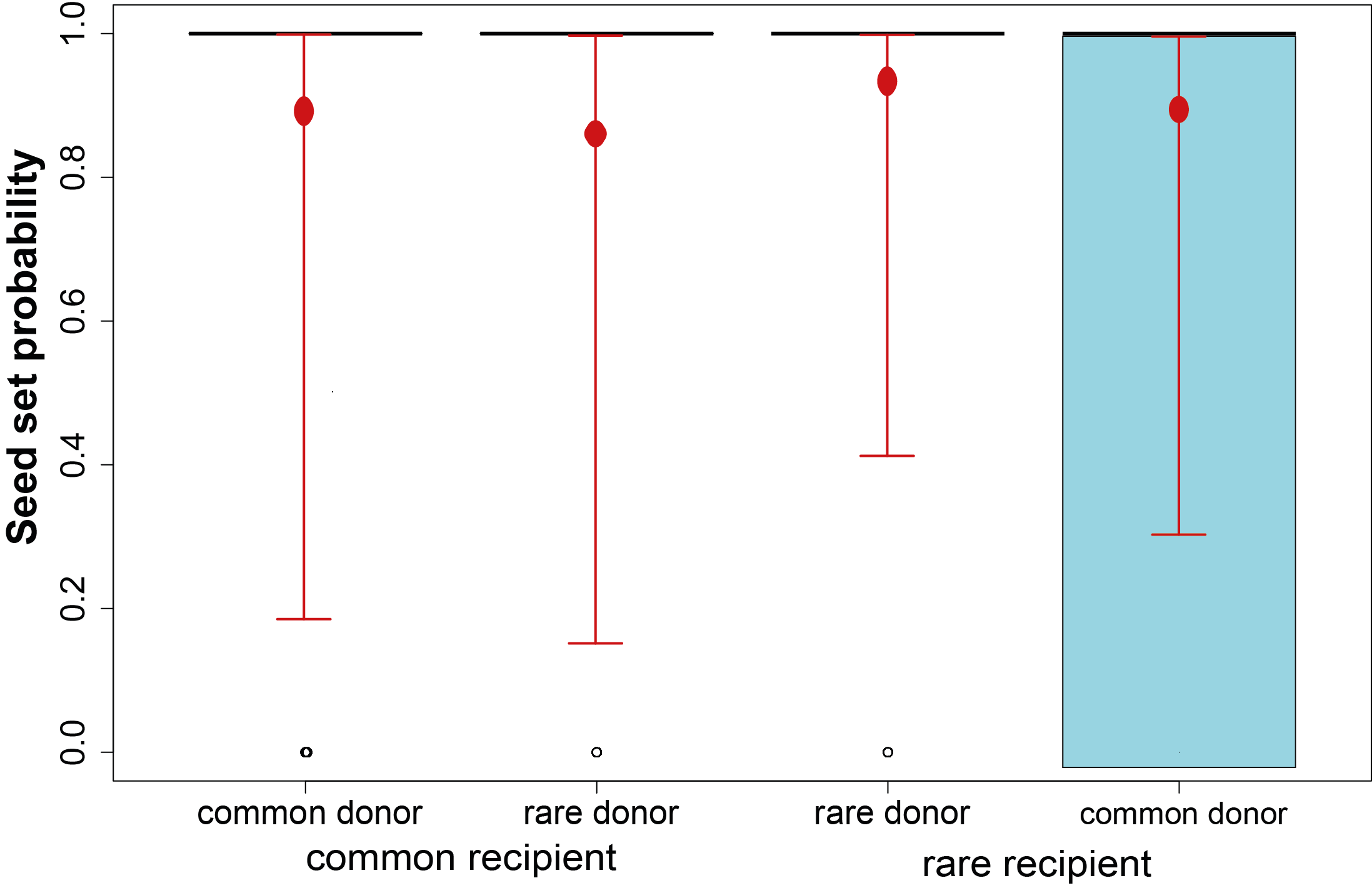


Figure S 4 Seed set probability for heterospecific treatment only for common and rare recipients with common and rare donors (dark blue: common recipient; light blue: rare recipient). The estimated marginal means with their 95% confidence intervals for each group are plotted in red on top of the boxplots of the raw data.


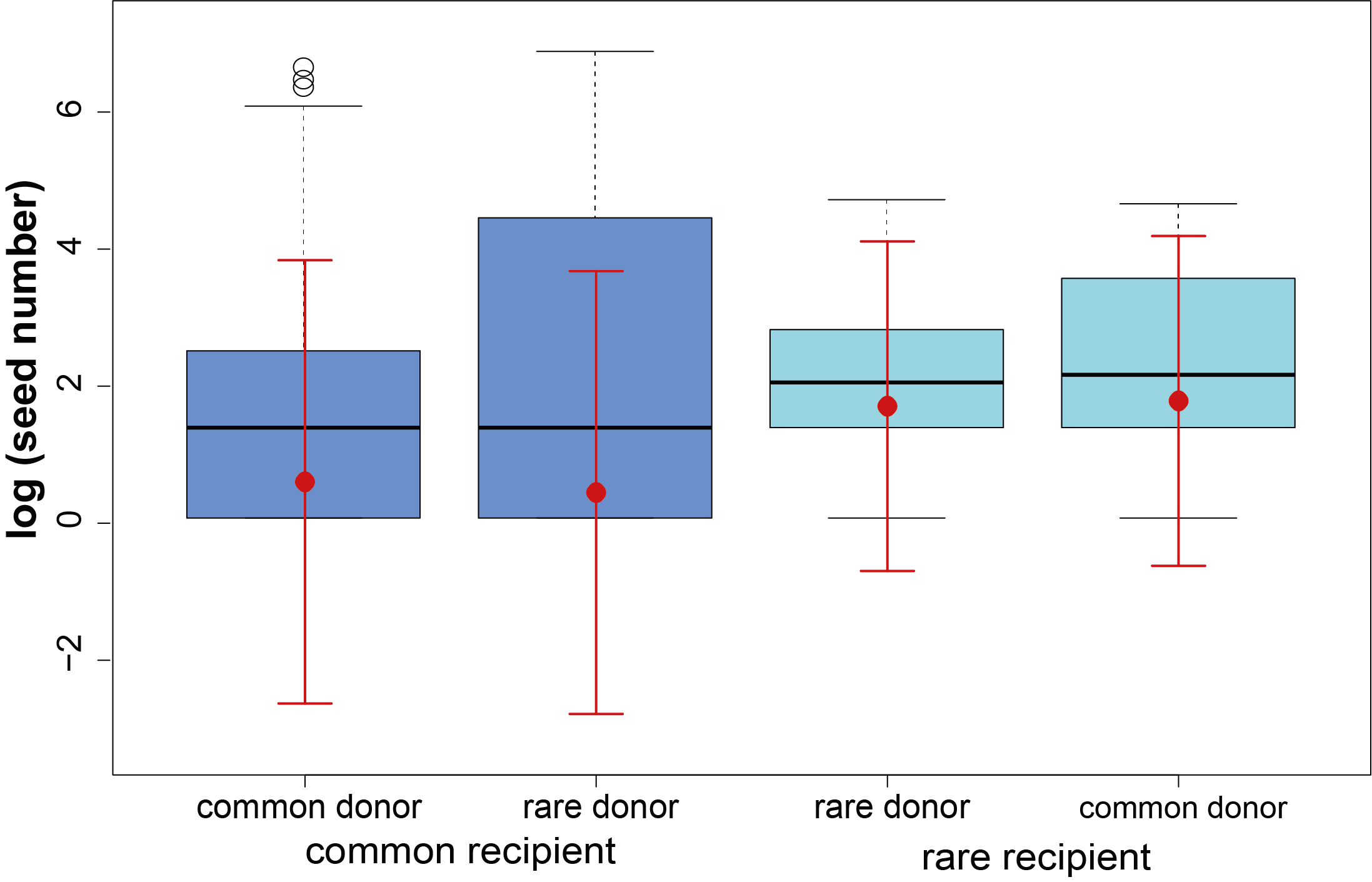


Figure S 5 Logged seed number for heterospecific treatment only for common and rare recipients with common and rare donors (dark blue: common recipient; light blue: rare recipient). The estimated marginal means with their 95% confidence intervals for each group are plotted in red on top of the boxplots of the raw data.


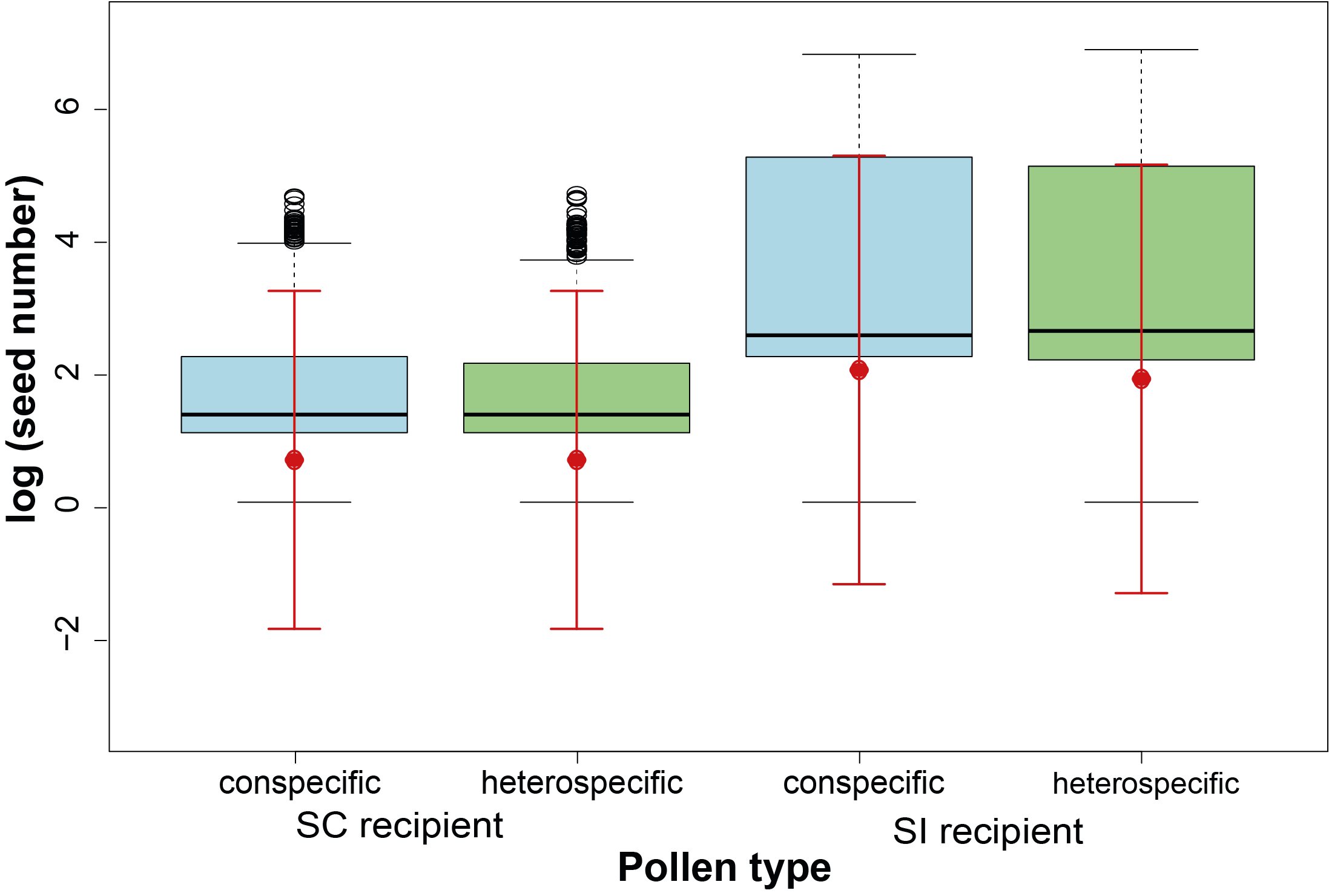


Figure S 6 Logged seed number for conspecific and heterospecific treatments for self-compatible (SC) and self-incompatible (SI) recipient species (light blue: conspecific treatment; light green: heterospecific treatment). The estimated marginal means with their 95% confidence intervals for each group are plotted in red on top of the boxplots of the raw data.


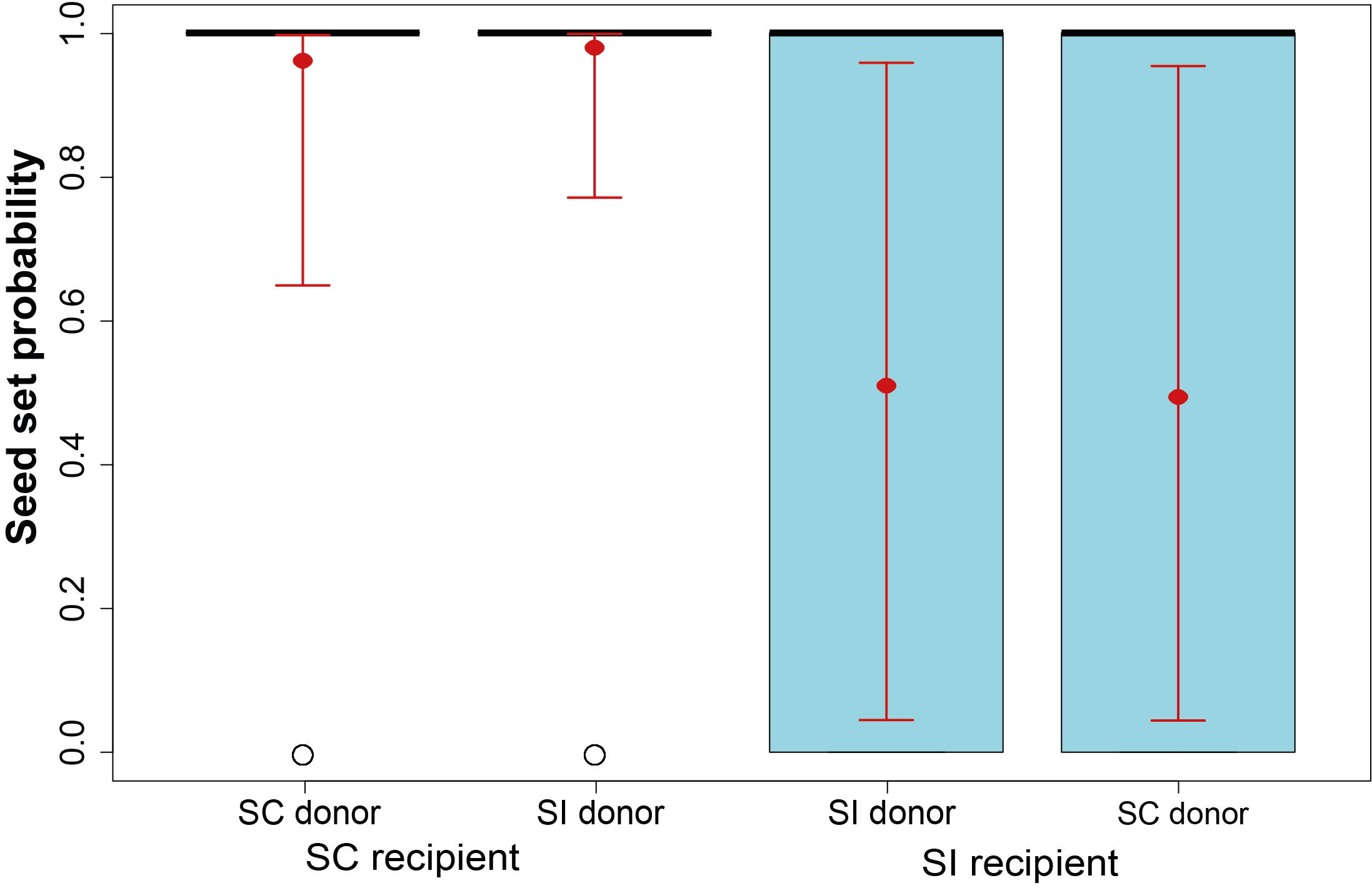


Figure S 7 Seed set probability for heterospecific treatment only for self-compatible and self-incompatible recipient species with self-compatible and self-incompatible donors (SC: self-compatible, SI: self-incompatible; dark blue: SC recipient; light blue: SI recipient). The estimated marginal means with their 95% confidence intervals for each group are plotted in red on top of the boxplots of the raw data.


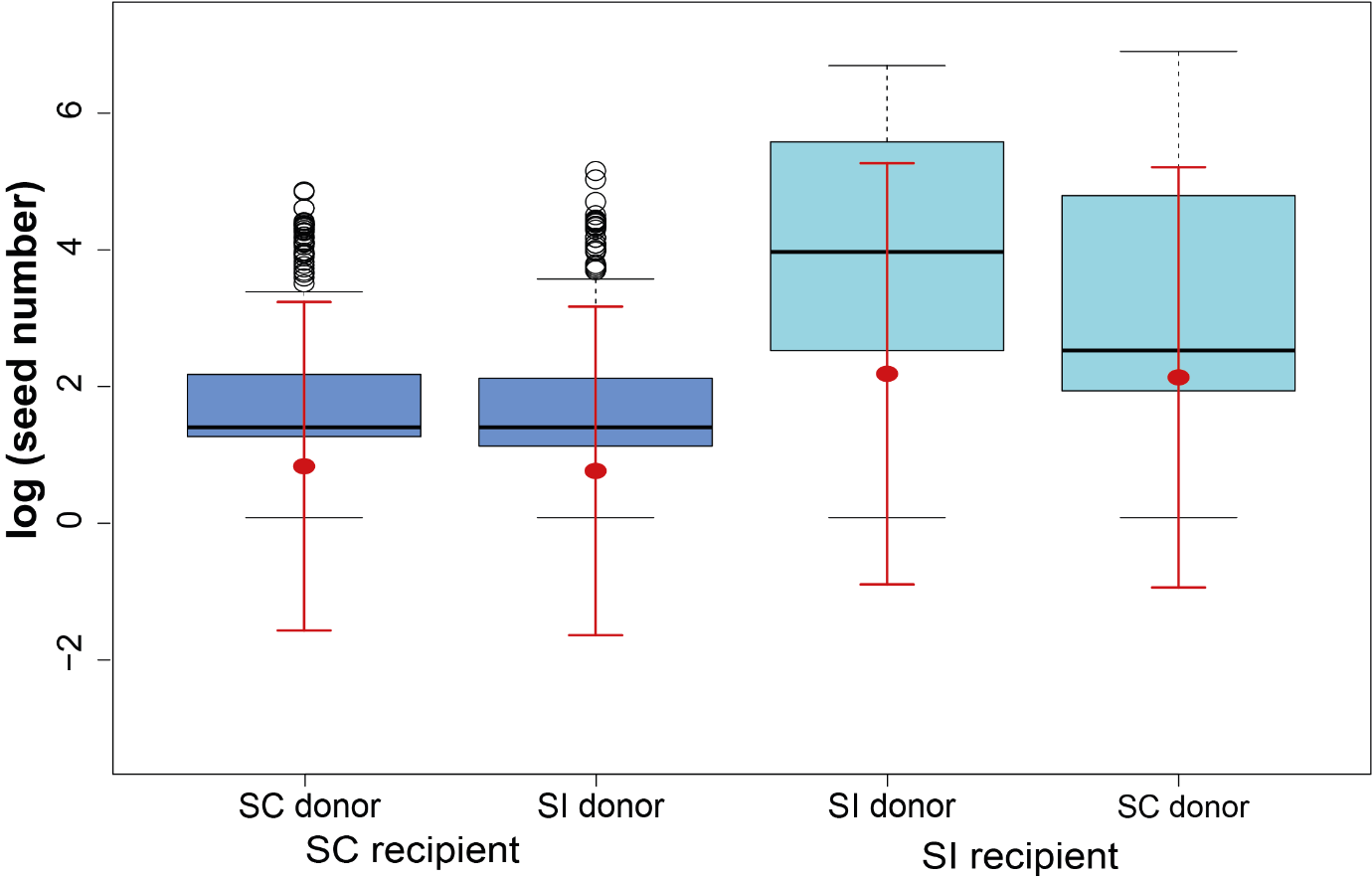


Figure S 8 Logged seed number for heterospecific treatment only for self-compatible and self-incompatible recipient species with self-compatible and self-incompatible donors (SC: self-compatible, SI: self-incompatible; dark blue: SC recipient; light blue: SI recipient).The estimated marginal means with their 95% confidence intervals for each group are plotted in red on top of the boxplots of the raw data.

Figure S 9 Log-response ratio of seed number with heterospecific treatment (HP) to seed number with conspecific treatment (CP), within each pair (same pair ID) for non-zero counts in relationship with the phylogenetic distance (PD) between recipient and donor species for a) common recipient with common donor b) common recipient with rare donor c) rare recipient with rare donor d) rare recipients with common donors. The confidence intervals for the estimated trends are plotted around the estimated relationship (dark blue: common recipient; light blue: rare recipient). Log-response ratio values < 0 correspond HP seed number < CP seed number (HPI present); values equal to 0 correspond to HP seed number = CP seed number (no HPI); values > 0 correspond to HP seed number > CP seed number (HP improves seed set)


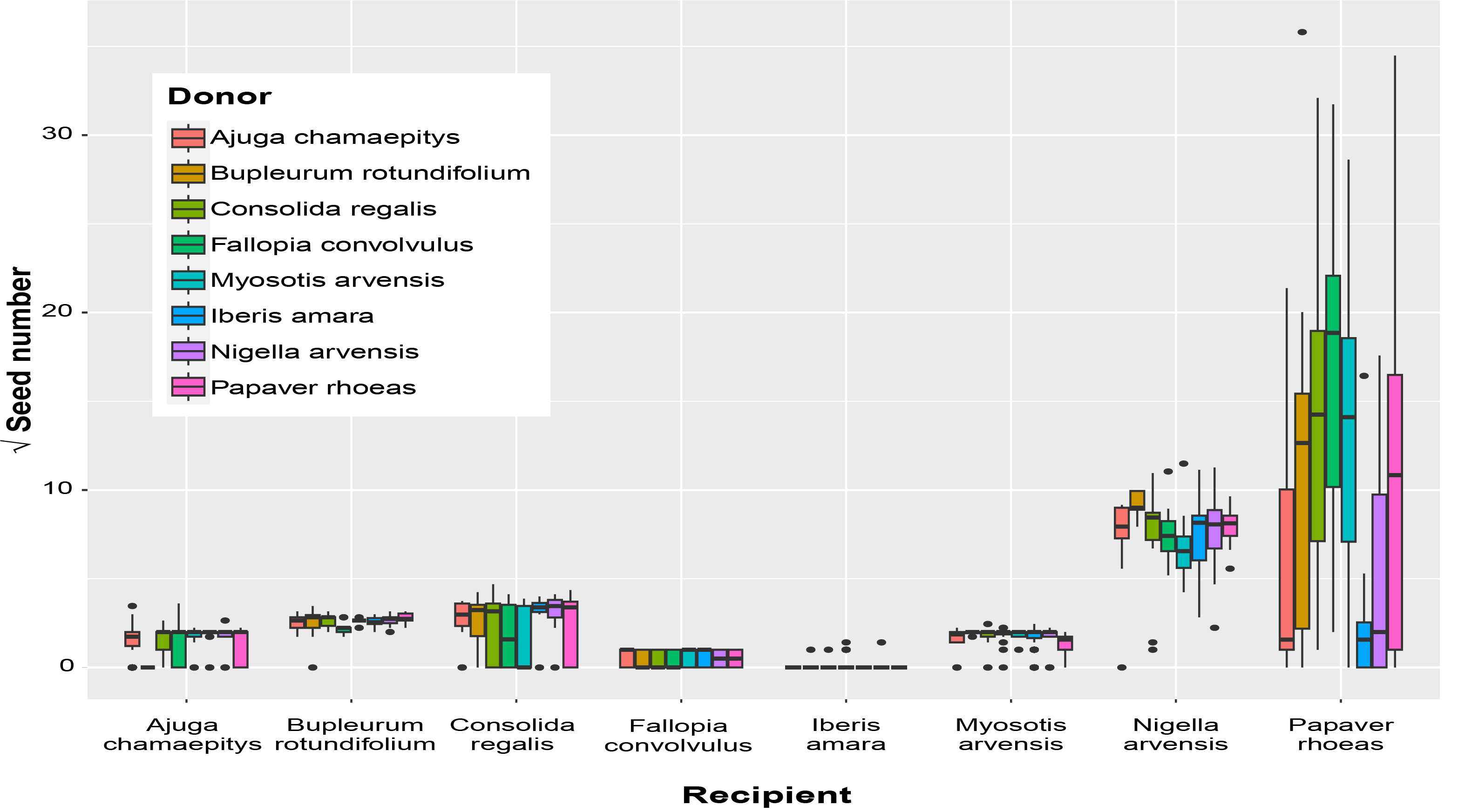
Figure S 10 Square-rooted seed number versus Donor-Recipient combination.

## SI Methods

### **Protocol seed counting in ImageJ**

**Photographing**:

Nikon D750 with a 60mm f/2.8 Macro lens (Nikon), cable release (in the photography equipment)

Kaiser Image Capturing set with height-adjustable column, 2x light unit.

The two light units were placed right and left of the column, set at a height of 4” (upper end), as close to the macro lens as possible. Lighting was set to the highest intensity. The camera was screwed on the plate and set to a height of 46” (upper end).

Camera settings: manual mode (M), f/11, ISO 100, shutter speed = 1/100 s. Images taken as JPG with finest quality. Focusing was done with the built-in autofocus (AF) and checked on the display using the magnifying option (+ sign). After focusing for the first capture, AF was switched off in order to keep the focus plane constant; it was occasionally checked throughout the photographing session. In order to know the ID of the seeds photographed, a tiny etiquette with the label written on it was placed in the corner for each image of each seed bag.

**Postprocessing**:

Images were transferred onto the PC/laptop, eventual duplicates were deleted (image with best lighting was kept), and the pictures were labelled accordingly to the etiquette in the image.

Images were then imported into Lightroom (version 6) in order to crop the image and, where necessary, remaining parts of the etiquette was removed using the “Bereichsreparatur” tool. The same was used in case some seeds would have been cut off by cropping the image, so the original seed was simply copied into the remaining image area before cropping.

Where necessary, image brightness was adjusted to improve contrast for the conversion to a black-white binary in ImageJ later. For the exporting, images were not resized and only standard level sharpening was applied.

**ImageJ**:

Open Image

Process > Binary > Make Binary # converts image into a black-white binary image

Process > Binary > Watershed # automat. separates seeds that might be overlapping

Image > Adjust > Threshold:


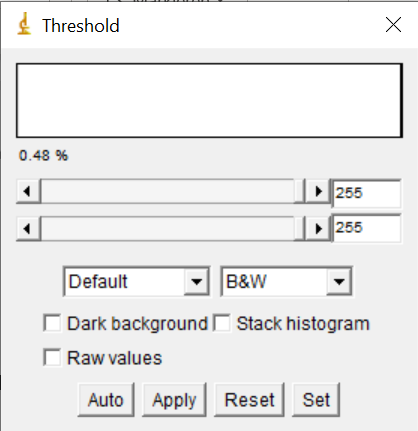


Analyze > Analyze Particles:


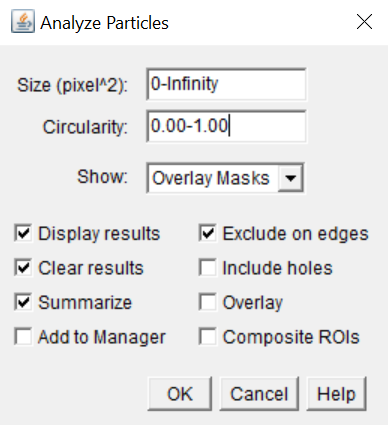


After some initial tests, following threshold have been defined:
Size: 150-399 for small seeds that are likely not fully developed

400-infinity for seeds that seem fully developed

Circularity: 0.65 – 1

For quicker analysis, a macro has been defined to automatically analyze particles in all 244 images.

In total, three passages have been done, with size ranges 150-399 (small), 400-infinity (large), and 150-infinity (full), respectively. This was done to allow for comparison if the sum of the two classes small + large = full, to check whether the macro worked correctly. After each passage, the results were exported into an excel file, where using the “ZÄHLENWENNS” function the particles in each size class were counted for each image. The used code for the macro is inserted hereafter:

// Macro to measure Area, Intensity, Perimeter, and Shape of directory of images

run("Clear Results"); // clear the results table of any previous measurements

// The next line prevents ImageJ from showing the processing steps during

// processing of a large number of images, speeding up the macro

setBatchMode(true);

// Show the user a dialog to select a directory of images

inputDirectory = getDirectory("folder_with_images");

// Get the list of files from that directory

// NOTE: if there are non-image files in this directory, it may cause the macro to crash

fileList = getFileList(inputDirectory);

for (i = 0; i < fileList.length; i++)

{

processImage(fileList[i]);

}

setBatchMode(false); // Now disable BatchMode since we are finished

updateResults(); // Update the results table so it shows the filenames

// Show a dialog to allow user to save the results file

outputFile = File.openDialog("Save results file");

// Save the results data

saveAs("results",outputFile);

function processImage(imageFile)

{

// Store the number of results before executing the commands,

// so we can add the filename just to the new results

prevNumResults = nResults;

open(imageFile);

// Get the filename from the title of the image that's open for adding to the results table

// We do this instead of using the imageFile parameter so that the

// directory path is not included on the table

filename = getTitle();

setOption("BlackBackground", false);

run("Convert to Mask");

run("Watershed");

//run("Threshold...");

setThreshold(255, 255);

//setThreshold(255, 255);

run("Convert to Mask");

run("Analyze Particles...", "size=150-399 circularity=0.65-1.00 display");

// Now loop through each of the new results, and add the filename to the "Filename" column

for (row = prevNumResults; row < nResults; row++)

{

setResult("Filename", row, filename);

}

close("*"); // Closes all images

}

Comments:

Blue marked text = specify the folder with the pictures to be analyzed

Yellow marked text = adjust size and circularity parameters to fit your needs

Text after // serves comment to the code lines that follow
